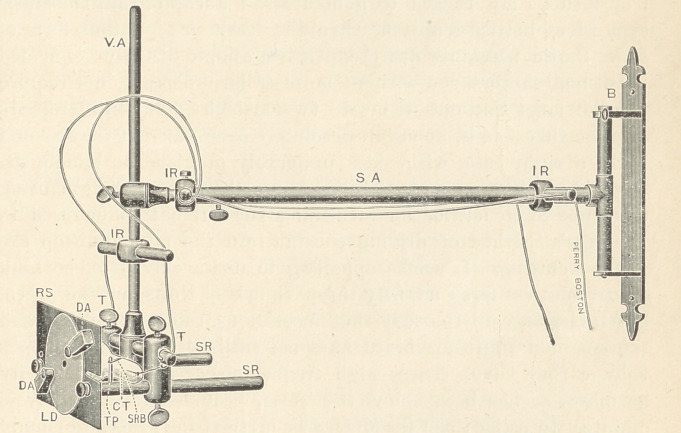# Dental Uses for Röntgen’s Discovery

**Published:** 1896-09

**Authors:** William Rollins

**Affiliations:** Boston, Mass.


### DENTAL USES FOR RÖNTGEN'S DISCOVERY.—SUPPORT FOR TUBE.

BY WILLIAM ROLLINS, BOSTON, MASS.

In using currents of fifty thousand volts, which are required for properly developing Röntgen rays, it is comforting to feel that the wires carrying the current cannot come in contact with the patient. To accomplish this I employ the bracket shown in the figure. B is the bracket plate fastened to the wall about five feet from the floor. To this is attached the swinging arm SA, movable horizontally. Inside this is another arm to which is attached the vertical arm VA bearing at its lower end the sliding rods SR, which are perforated on their upper sides with rows of holes into which fit the movable tube-pins TP, of hard rubber, which can thus be made to hold tubes of several forms by means of the small elastic bands SRB stretched over the pins. To the ends of the arms SR is attached the hard rubber shield RS, which prevents the patient from coming in contact either with the terminals T, T, or with the Crookes tube CT. This rubber shield has a three-inch hole in the centre, which is partly closed by an iron diaphragm, LD, coated with lead. The diaphragm is easily removable to allow others with different sized holes to be used. This arrangement allows the object looked at to be seen dimly over a considerable area and illuminates brightly that special part which is held against the hole in the diaphragm. The diaphragm is simply a form of Zentmeyer's microscope stage. It is freely movable in order to bring the opening opposite any part of the bulb of the Crookes tube, and is held in any position by the two arms DA. The rubber rods SR are movable in two horizontal directions and in vertical arc, this latter motion being required where two or more tubes are used at the same time, a plan which gives more light but an imperfectly illuminated field. Where two or more tubes are used it is well to use a generator for each, as the attempt to run more than one tube from the same generator gives less perfect results. In regard to generators, I am sure that the static type gives better definition than the Tesla or any other form of coil. Static machines are inexpensive, and, if directly connected with small motors, run without noise or jar. They do not require to be of large size, one with two twenty-inch plates will properly excite a tube. It is, however, absolutely essential that they should be in a tight case, with sides preferably of glass, in which the atmosphere is kept dry. This is particulary important in using this type of generator for furnishing the current to be used in McGraw's method of driving cocaine into the teeth to stop the pain of cutting. It seems appalling to use a current of so high electro-motive force as fifty-three thousand volts on the teeth, but this is due to the fact that most of us, though familiar with the common static machines, have not realized what an enormous voltage they have. These high frequency currents are perfectly harmless, as has been shown by Tesla, provided the quantity is minute. In using them for McGraw's method the generator should be so small that the maximum current it can be made to produce is a small fraction of a milliampère. This is a better method than using a larger generator with a shunt circuit. I shall figure a generator for this use and one for exciting a Crookes tube in the October number.

## Figures and Tables

**Figure f1:**